# Human Hepatocellular Carcinoma Classification from H&E Stained Histopathology Images with 3D Convolutional Neural Networks and Focal Loss Function

**DOI:** 10.3390/jimaging9020025

**Published:** 2023-01-21

**Authors:** Umut Cinar, Rengul Cetin Atalay, Yasemin Yardimci Cetin

**Affiliations:** Graduate School of Informatics, Middle East Technical University, Ankara 06800, Turkey

**Keywords:** hyperspectral imaging, hyperspectral microscopy, human carcinoma detection, hepatocellular carcinoma detection, cancerous cell classification, deep learning, convolutional neural networks, 3D convolutions, focal loss function, 3D-CNN

## Abstract

This paper proposes a new Hepatocellular Carcinoma (HCC) classification method utilizing a hyperspectral imaging system (HSI) integrated with a light microscope. Using our custom imaging system, we have captured 270 bands of hyperspectral images of healthy and cancer tissue samples with HCC diagnosis from a liver microarray slide. Convolutional Neural Networks with 3D convolutions (3D-CNN) have been used to build an accurate classification model. With the help of 3D convolutions, spectral and spatial features within the hyperspectral cube are incorporated to train a strong classifier. Unlike 2D convolutions, 3D convolutions take the spectral dimension into account while automatically collecting distinctive features during the CNN training stage. As a result, we have avoided manual feature engineering on hyperspectral data and proposed a compact method for HSI medical applications. Moreover, the focal loss function, utilized as a CNN cost function, enables our model to tackle the class imbalance problem residing in the dataset effectively. The focal loss function emphasizes the hard examples to learn and prevents overfitting due to the lack of inter-class balancing. Our empirical results demonstrate the superiority of hyperspectral data over RGB data for liver cancer tissue classification. We have observed that increased spectral dimension results in higher classification accuracy. Both spectral and spatial features are essential in training an accurate learner for cancer tissue classification.

## 1. Introduction

In 2020, approximately 960,000 new liver cancer cases were diagnosed, and 830,000 deaths due to liver cancer have been reported. Liver cancer is the sixth most encountered cancer type and is the third leading cause of cancer death globally. Hepatocellular carcinoma (HCC) is the most common type of liver cancer, with an occurrence rate of 80% [1]. In 70–90% of HCC patients, the risk factors include hepatitis B virus, hepatitis C virus, exposure to aflatoxin B1, cirrhosis, and excessive alcohol consumption. The tumor nodules can be monitored with ultrasonography at the early stages, and resection is considered the primary treatment method for patients with sufficient liver functionality and small tumor solitary scores. For more complex cases, treatment procedures might include liver transplantation, chemoembolization, and molecular-targeted therapies [2]. Pathology is an essential field for diagnosing HCC, screening the grade of disease, detecting the risk of recurrence after surgery, and developing new treatment techniques, including medicines. A variety of genetic mutations occur within the cells during cancer disease. The cancerous tissues are differentiable by expert pathologists with the help of a biopsy operation. However, the tissue examination process is tedious and time-consuming for expert pathologists [3].

A significant number of researchers have worked on computer-aided diagnosis (CAD), including the tumor detection problem for many years [4]. For the problem of detecting cancerous tumors from pathological images, there are various methods proposed. Using RGB data, the study [5] suggests using Atrous Spatial Pyramid Pooling (ASPP) blocks to obtain multi-scale texture features from Hematoxylin and Eosin (H&E) stained HCC histopathology images. ASPP blocks have been placed after each max-pool layer to generate a multi-scale sample space. With the help of this approach, texture features in images are effectively utilized by the deep neural network, and the experimental results showed 90.93% accuracy in the four-category classification of HCC images. Another study [6] works on the HCC grade differentiation from multiphoton microscopy images. The researchers adapt VGG-16 CNN topology to train a classifier on a dataset consisting of three grades of HCC disease, including well-differentiated, moderately-differentiated, and poorly-differentiated groups. Over 90% HCC differentiation accuracy has been obtained, and the results show the validity of deep learning approaches with multiphoton fluorescence imagery. Alternatively, hyperspectral imaging is a powerful tool for describing the subject matter up to its chemical properties, and it has many applications in remote sensing [7], agriculture [8], food safety [9], environment [10], and more [11]. Hyperspectral imaging technology provides promising results for CAD researchers [12]. A 2001 study shows an early example of hyperspectral imaging integrated with a light microscope [13]. A reference hardware system is devised to capture hyperspectral cubes from microscopy tissue slides. The proposed approach combines an imaging spectrograph with an epi-fluorescence microscope. Different wavelength light sources have been used to illuminate the samples, such as a 532 nm solid-state laser, a helium-neon laser, an argon ion laser, and a pulsed-doubled nitrogen dye laser. The sample slide is moved with a motorized mover during the data capturing. A Charge-Coupled Device (CCD) camera captures the reflected light, and custom software is developed to visualize and store hyperspectral data. The study [14] proposes a new approach based on hyperspectral image analysis to address the Anaplastic Lymphoma Kinase (ALK) positive and negative tumor identification problems. Sixty-channel hyperspectral data from lung cancer tissues are captured by an Acousto-Optic Tunable Filter (AOTF) based hyperspectral imaging system. Using a Support Vector Machine (SVM) based segmentation algorithm, the lung tissue images are segmented into three regions: cell nucleus, cytoplasm, and blank area. The accuracy of the segmentation model is calculated with the help of manual ground truth data provided by a lung cancer expert. The segmentation accuracy for each class is evaluated to conclude a treatment prescription focusing on ALK-positive and ALK-negative tumor diversity. In another study [15], a similar hyperspectral imaging system powered by AOTF is employed to collect hyperspectral data from bile duct tissue samples with 30 channels. Deep convolutional neural networks (CNN) with architectures Inception-V3 [16] and Restnet50 [17] are deployed for building a prediction model. A spectral interval convolution method is proposed to adapt hyperspectral data with deep learning architectures. CNN experiments have been conducted by feeding image patches to the network. A random forest-based approach is utilized to provide scene-level predictions by combining image patch predictions from the same scene. The authors have reported a tumor detection accuracy of 0.93 with hyperspectral data and 0.92 with RGB data.

This paper proposes a new HCC tumor detection framework based on hyperspectral imaging and 3D Convolutional Neural Networks. We have built a microscopy biological tissue image-capturing system in-house by integrating a push broom VNIR hyperspectral camera and a light microscopy device. We collected a wide range of spectral data between 400 nm to 800 nm from liver tissue samples. The captured images from each sample are divided into smaller patches and fed into a custom 3D convolution-based CNN learner to generate a strong cancer tissue prediction model. With 3D convolution operation, both spectral and spatial features are considered while training the classification model. 3D convolution operation enables the capture of local spectral features in the hyperspectral cube. Furthermore, we have employed the focal loss function as the CNN cost function to overcome the class imbalance problem [18]. We have empirically demonstrated that 3D convolutions significantly improve classification accuracy compared to 2D convolutions operated on the same dataset. During our experiments, we demonstrated the superiority of hyperspectral data over its RGB counterpart.

Compared to the existing literature, we employ more spectral bands for the tissue classification task in this study. Although AOTF is a widely used piece of equipment in tissue classification tasks, there are studies reporting that AOTF may not be reliable enough for radiometric measurements due to the lack of homogeneity of the diffraction efficiency [19,20,21]. Unlike the existing studies, we employ a hyperspectral VNIR camera as primary imaging equipment to obtain reliable data. We introduce a solid classification framework based on a new deep-learning topology and 3D convolutions. Additionally, in contrast to other studies, we clearly compare performances of hyperspectral, Principal Component Analysis (PCA) of hyperspectral and RGB datasets on classification accuracy. The contributions of this paper can be summarized as follows. Firstly, we have built a biological tissue image capture system in our laboratory by integrating a hyperspectral camera and a light microscope with a 3D-printed motorized stepper. Secondly, we have demonstrated that hyperspectral data considerably improves classification performance. RGB data can represent spatial features of tumor tissues in fine detail, while, hyperspectral imaging captures both spatial and spectral features of tumor tissues leveraging the deep neural network classification accuracy. Thirdly, our proposed method takes advantage of the hyperspectral cube by utilizing 3D convolutional neural networks. 3D kernels enable the learner to extract voxel information with a compact approach. The use of a 3D convolution operator in CNN can generate both spectral and spatial features via the same single convolution operation. Additionally, our method does not require manual feature engineering as pre-processing or post-processing stages in the classification pipeline. Thus, 3D convolutional neural networks commit better generalization performance with a simpler network topology. Finally, our paper tackles the class imbalance problem, a common challenging aspect for most medical image analysis studies. We have employed the focal loss function within our classification model. The focal loss method compensates for class imbalance by using a focusing parameter in the cross-entropy function, and the learner’s sensitivity to misclassified samples is boosted. Furthermore, the focal loss function is capable of increasing model generalization without causing overfitting.

The rest of the paper is organized as follows. In Section 2, we give details of our methodology, including data capture and deep learning steps. Section 3 presents our experimental results by comparing different sets of parameters and learner configurations. Finally, in Section 4, we provide discussions with a brief conclusion of the study, including its limitations and suggestions for future research.

## 2. Materials and Methods

### 2.1. Data Acquisition

**Figure 1:**
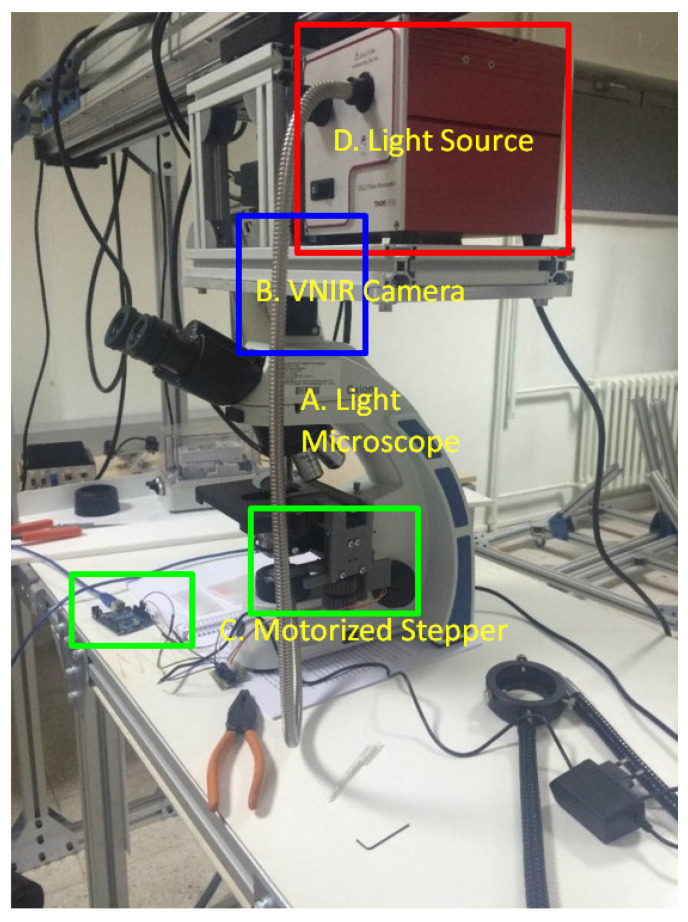
Data Acquisition system. A, Light microscope; B, VNIR Camera; C, Motorized Stepper; D, Light Source.

**Figure 2:**
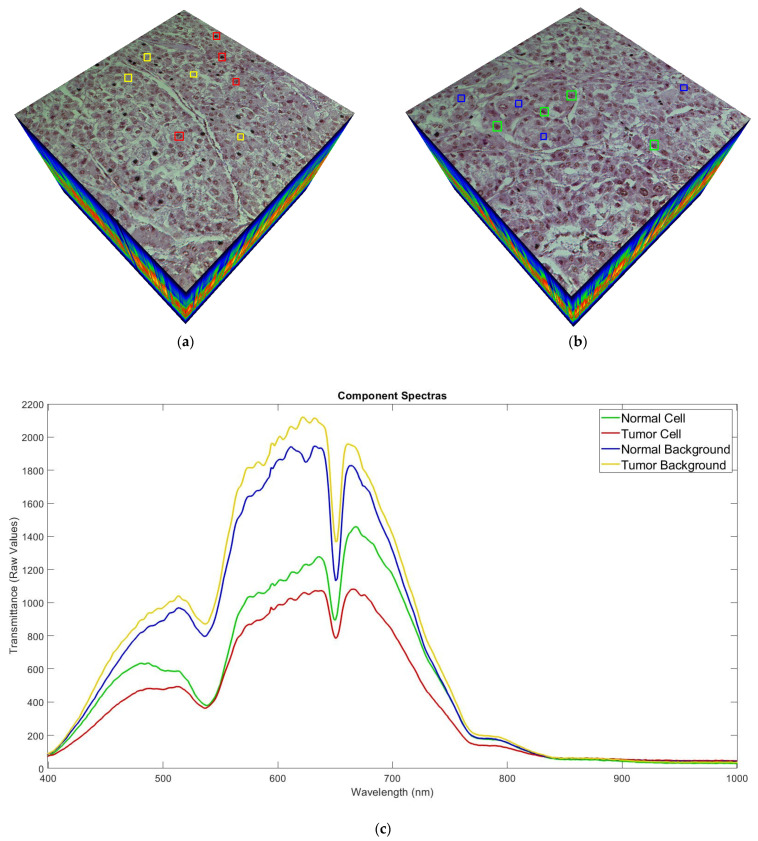
Hyperspectral tissue samples dataset. (**a**) Tumor (hepatocellular carcinoma, HCC) tissue sample, tumor cells (red), tumor background tissue (yellow); (**b**) Normal (Healthy) tissue sample, normal cells (green), normal background tissue (blue); (**c**) Spectra comparison plotting of the given components.

In this study, we have developed a hyperspectral microscopy image-capturing system by integrating a Headwall A-series VNIR model push-broom hyperspectral camera (Headwall Photonics Inc., Bolton, MA, USA) and a Euromex Oxion light microscope (Euromex, Arnhem, The Netherlands) in our laboratory. A sample photograph from our data acquisition system is presented in Figure 1. The light microscope’s objective lens is configured to display the samples with 40× magnification. The hyperspectral camera is capable of capturing 408 spectral bands between 400 nm to 1000 nm. We calibrated and verified our imaging setup using a microscope stage calibration slide for optimum image quality. In this regard, our imaging system’s spatial resolution is 0.55 microns. Our imaging system measures a liver cell nucleus around 12–18 pixels in diameter, and 6.6 to 9.9 microns, which is also correlated with the clinical measurements of the human liver cell size [22]. In addition to hyperspectral images, the camera simultaneously captures RGB images of the same scene. To capture data with the proper geometry from our hyperspectral camera, we have devised a motorized moving table hardware solution to gradually move tissue samples while the camera is in capture mode. The motor speed is controlled by a small Arduino device (Atmel Corporation, San Jose, CA, USA), which is optimized to capture tissue sample images with the highest resolution along the track direction. The tissue samples are illuminated from the bottom by a 12 V, 100 W halogen light source (Thorlabs, Newton, NJ, USA). All images are captured in a dark room without light sources other than the halogen lamp placed at the bottom of the tissue slide. For radiometric calibration, we have collected white references from the empty glass slide illuminated by the halogen lamp as in the regular capture mode. In addition, we have collected dark references by blinding the camera sensor with its lens cap. An example of captured data from healthy and unhealthy classes and corresponding tissue components, including cell and background, can be seen in Figure 2. The spectra sketches in Figure 2 are obtained by obtaining the area average of the selected regions from the sample image captured with 40× lens magnification. It can be inferred that normal and tumor cell samples transmit different spectral signatures for their particular components.

By morphologically inspecting the tissue spectra in Figure 2, we see two noticeable dips around 540 nm and 650 nm. Eosin in H&E staining has a very characteristic dip around 540 nm [23,24]. In fact, those two dips are compatible with previous findings from previous studies [25,26] working on hyperspectral data of liver tissue samples.

### 2.2. Classification

Hyperspectral imaging provides a high potential for classification tasks when both spectral and spatial data are fused inside a machine learning model. However, the machine learning applications developed with a hyperspectral imaging base might be prone to overfitting the training data due to high dimensionality. In fact, for small datasets, complex classifiers like CNN and SVM tend to overfit by learning random noise in the data instead of extracting generative relations between the classes [27]. In addition, manual feature engineering operations on the dataset can significantly reduce the trained model’s generalization capability. Manually crafted features restrict the feature space for the classifier, whereas deep learning models can automatically find optimal features and extract indirect and nonlinear relationships between features. Therefore, in this study, we aim to develop a fully automatic classification model with high generalization capability on the HCC detection problem.

To fully exploit the effectiveness of automatic feature learning in deep learning, we employed a CNN-based learner using 3D convolutions. 3D-CNN models are commonly used in 3D object recognition [28], video action recognition [29], and medical image recognition [30] studies. 3D-CNN learners commit to the effective utilization of spatial-spectral data and high generalization performance for hyperspectral data. Thus, spectral signature information encoded within a 3D hyperspectral cube is extracted together with the textural information available on the spatial plane.

The main difference between traditional 2D-CNN and 3D-CNN is the mechanics of convolution operation applied at the convolution layers. The kernel slides along two dimensions (*x* and *y*) on the data in 2D-CNN classifiers while, in 3D-CNN classifiers, the kernel slides along three dimensions (*x*, *y*, and *z*) on the data. 3D-shaped kernels used in convolutions can describe the features in spatial and spectral directions. In addition to spatial features like texture and shape attributes, the spectral dimension can be embedded in the final classification model to capture radiometric information. We employed the 3D convolution operation proposed in the study [29].

(1) $$v_{ij}^{xyz}=f\left(\underset{m}{\sum }\overset{P_{i}-1}{\underset{p=0}{\sum }}\overset{Q_{i}-1}{\underset{q=0}{\sum }}\overset{R_{i}-1}{\underset{r=0}{\sum }}w_{ijm}^{pqr}v_{\left(i-1\right)m}^{\left(x+p\right)\left(y+q\right)\left(z+r\right)}+b_{lj}\right)$$

Mathematically, 3D kernels can be formulated as in Equation (1), where $v_{lij}^{xyz}$ represents the value at position (x,y,z) in the jth feature map in the ith layer, m is the index value of the input feature maps from the (i−1)th layer connected to the current feature map, $P_{i}$, $Q_{i}$ and $R_{i}$ are the height, width, and depth of the kernel, respectively, $w_{ijm}^{pqr}$ is the kernel value at position (p,q,r) for $m^{th}$ feature map in the previous layer, b is the bias, and f(.) is the activation function.

As an activation function, a non-saturating Rectified Linear Units (ReLU) function is used as proposed in [31]. The formulation of the ReLU activation function is given as

(2) f(v)=max(0,v)

**Figure 3:**
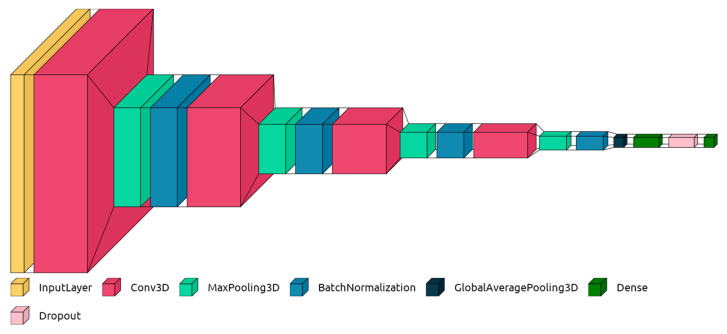
3D-CNN topology sketch.

We have designed a custom CNN topology according to the details given in Table 1 and illustrated in Figure 3. In the network, there are max-pooling layers defined between the consecutive convolution layers to decrease the number of parameters and the complexity of the model [32]. Furthermore, a batch normalization layer follows each max-pooling layer to reduce internal covariate shift. The batch normalization layer also helps to speed up training by applying a normalization so that the mean value is around 0 and the standard deviation is 1. Hence the learner can utilize a larger learning rate in the optimizer algorithm. Furthermore, instead of a conventional fully connected layer, we have employed a global average pooling layer to generate feature maps into a 2D structure before feeding to the final dense layer. As elaborated in the paper [33], the global average pooling layer is not prone to overfitting since it has no parameter to optimize. It is also invariant to spatial translations in the input since it amounts to spatial averaging. In this way, we can simultaneously tackle overfitting due to the structure of texture features in our training set and eliminate the effect of noise caused by the tiny vibrations in the stepper motor.

For convolution kernel size, we have selected 3 × 3 × 3 following the best practice suggested by [34]. For CNN training, we used the Adam optimizer, as proposed in [35], with default parameters (β_1 = 0.9 and β_2 = 0.999) a and learning of rate 0.001. We set the batch size to 128, trained the models for 100 training epochs, and used a 10% of dropout rate.

As in most medical studies [36], our dataset is imbalanced due to the presence of very few healthy compared to tumor samples. Therefore, CNN classifiers can be biased towards the majority class and may result in false positives in medical applications. We have employed the focal loss (FL) function to overcome the class imbalance problem in our dataset. Traditionally, the cross entropy (*CE*) function is employed in most deep learning models.

(3) $$CE\left(p_{t}\right)=-\log\left(p_{t}\right)$$

where $p_{t}$ is given by

(4) $$p_{t}=\{\begin{array}{c} p\;\;\;\;\;\;\;\;\;y=1\;\\ 1-p\;\;\;\;\;\;\;\;y=-1 \end{array}$$

where y∈{−1,1} is the ground truth class and p∈[0,1]  is the classifier’s output probability value for the class y=1.

Nevertheless, in case of extreme class imbalance, the loss contribution of well-classified examples in cross-entropy-based models can easily dominate the minority class. The balanced cross entropy (BCE) function, as defined in Equation (5), is employed for dealing with the class imbalance problem in the traditional CE function.

(5) $$CE\left(p_{t}\right)=-a_{t}\log\left(p_{t}\right)$$

where $a_{t}$ is a weighting factor hyperparameter and defined as

(6) $$a_{t}=\{\begin{array}{c} a\;\;\;\;\;\;\;\;\;if\;y=1\;\\ 1-a\;\;\;\;\;\;\;\;otherwise \end{array}$$

where a ∈[0,1]. The BCE function helps to balance the contribution of minority and majority classes during the training. However, it does not affect the loss between easy/hard examples. Our dataset contains an extreme imbalance, the easy positives (tumor samples with high $p_{t}$) can dominate the training and cause too much focus on easy positives. The focal loss function, however, can down-weight the loss contribution of easy examples and relatively increase the loss contribution from the hard examples. Focal loss is derived from the cross-entropy loss function (3) by introducing a modulating factor ${\left(1-p_{t}\right)}^{\gamma }\;$ to the cross-entropy loss. In this study, we have employed a balanced version of the focal loss function, defined in Equation (7).

(7) $$\mathrm{FL}\left(p_{t}\right)=-a{\left(1-p_{t}\right)}^{\gamma }\;\log\left(p_{t}\right)$$

where γ≥0 is the focusing parameter. The weighting factor, a, enforces the training procedure so that the learner concentrates on the minority class instead of treating the classes with equal importance. At the same time, the focusing parameter, γ, imposes focusing on the examples resulting in large errors, namely hard examples [18].

## 3. Results

### 3.1. Dataset

In this study, we employed a liver tissue array from Biomax LV962 (TissueArray.Com LLC, Derwood, MD, USA), a commercially available H&E-stained liver tissue slide. The tissue microarray contains both healthy and unhealthy cases; 3 normal liver tissues, 1 cancer adjacent liver tissue, 1 each of metastatic adenocarcinoma and cavernous hemangioma, 4 liver cirrhosis, 3 cholangiocarcinoma, and 32 hepatocellular carcinoma. From each case, our dataset contains two tissue samples. We have employed normal (healthy) and hepatocellular carcinoma (unhealthy) classes from the tissue microarray. As depicted in Table 2, there are 6 healthy and 54 unhealthy tissue samples in our dataset.

We have evenly divided the dataset into three subsets, including training, validation, and testing sets. Therefore, all three sets include distinct patients and there is no overlap between them.

**Figure 4:**
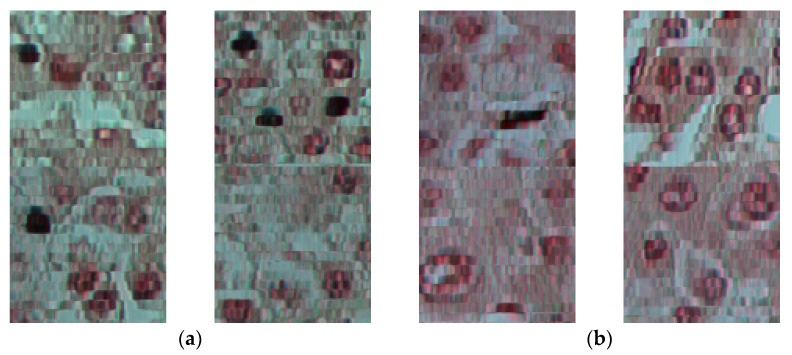
Sample patch images taken with 40X magnification, and the image size is 100 × 100 pixels. (**a**) Tumor sample patches; (**b**) Healthy sample patches.

In the dataset, each sample image is captured with 1000 × 2000 pixels resolution and 40× microscopy lens magnification. As shown in Figure 4, for visualization purposes, we have generated an RGB representation from the hyperspectral cube by fitting three normal distributions synthesizing red, green, and blue bands with a standard deviation of 25 and mean values of 630, 540, and 480 respectively. Sample images are divided into smaller patch images with size S×S pixels, where we took S as a parameter. In some patches, there were blank areas without any tissue samples. The image patches with more than 50% blank area were automatically removed from the dataset to obtain a reliable dataset, and 4% of the data was eliminated with this method. Our hyperspectral imaging system can output 408 bands between 400 and 1000 nm. However, manually inspecting the samples, we have observed that the bands above 800 nm contain a low signal-to-noise ratio. Therefore, we have only used the first 270 bands between 400 and 800 nm to reduce computational cost and prevent flawed information from being presented to the classifier.

### 3.2. Hardware and Software Configuration

We have employed an AI server with eight NVIDIA V100 Tensor Core 32GB GPUs with 5,120 Tensor Cores, delivering up to 1 petaflop of AI computing performance. The server machine has a dual Intel Xeon E5-2620 v3 CPU and 128 GB of DDR4 memory. By using this server, eight distinct models can be trained simultaneously. The software stack used in our study includes Python 3.8, Keras 2.3.1 with Tensorflow 2.0 for deep learning programming, CUDA for GPU acceleration, and Ubuntu 18.04 for the main operating system.

### 3.3. Evaluation Metrics

We have employed accuracy, precision, recall, and F1 score metrics formulated in Equations (8) to (12) to evaluate the classification performance. Moreover, we have used the Matthews Correlation Coefficient (MCC) metric, which is generally suggested for the classifiers focusing on class imbalance problems in medical studies [37] as formulated in Equation (9). The output value of the MCC metric varies between −1 and 1, such that 1 represents a perfect prediction, 0 means a random prediction, and −1 implies total disagreement between prediction and observation. All metrics are calculated from the classifier output metrics including True Positive (*TP*), True Negative (*TN*), False Positive (*FP*), and False Negative (*FN*).

(8) $$Accuracy=\frac{TP+TN}{TP+TN+FP+FN}$$

(9) $$Precision=\frac{TP}{TP+FP}$$

(10) $$Recall=\frac{TP}{TP+FN}$$

(11) $$F1Score=2\cdot \frac{precision\cdot \;recall}{precision+recall}$$

(12) $$MCC=\frac{TP\cdot TN-FP\cdot FN}{\sqrt{\left(TP+FP\right)\left(TP+FN\right)\left(TN+FP\right)\left(TN+FN\right)}}$$

### 3.4. Experimental Results and Discussion

To evaluate the performance of the proposed method, we trained distinct CNN classifiers with different configurations but the same topologies as depicted in Table 1. We have empirically found the optimal values for image patch size S. As stated in [18], the hyperparameters γ and α in the focal loss function are dataset-specific and need to be tuned for different model and dataset configurations. Therefore, in order to provide a fair evaluation, we optimized focal loss hyperparameters for each configuration. Furthermore, we have compared the classification performances of different spectral resolutions such as hyperspectral, sampled hyperspectral, PCA of hyperspectral, and RGB. Afterward, to reveal the effect of kernel dimensionality (2D vs. 3D kernels), we experimented with the implications of convolution operation by comparing 2D and 3D convolution-based CNN results. Finally, we have conducted another experiment by rotating our dataset splits between training, validation, and testing subsets to ensure that our models are not overfitting on the data.

In the first experiment, we explored the impact of patch size (S) on classification performance. The patch size is an important parameter for our classification method since it determines the amount of variation in textural features on a single patch image. The textural features are composed of different components such as cell nucleus, cytoplasm, and blank area in the tissue sample. Therefore, the size of the cropped patches should not be too small to miss important textural features. Similarly, the classifier might tend to only focus on dense areas when the size parameter is too large. For this purpose, we have conducted experiments using four different values for patch size parameters as given in Table 3. We obtained the best classification accuracy and MCC value with a 100 × 100 pixels patch size. For the remaining experiments, we have fixed the patch size parameter to 100.

In the second phase of the experiments, we have identified the optimal focal loss hyperparameters, *γ* and *α*, for the HSI dataset. We have compared the classification performance of the balanced cross-entropy function with the focal loss function. In the focal loss cost function, the weighting factor, *α*, enables the loss function to output differentiated loss values for the minority (healthy) and majority (tumor) classes. It balances the influence of negative and positive examples on the loss. Meanwhile, the focusing parameter, *γ*, effectively reduces the loss contribution from well-classified, namely, easy, examples while keeping the high loss contribution of hard examples. This way, the focusing parameter, *γ*, adjusts the level of focus on the hard examples during the training stage. The focusing parameter value should be tuned to deal with the misclassified hard examples while maintaining the overall classification accuracy and MCC score. The optimal values for the focal loss hyperparameters, *α* and *γ*, depend on the severity of the imbalance and the existence of hard and easy examples in the dataset. Hence, the optimal values depend on the dataset. As stated in the paper that first introduced the focal loss function [18], the gain in modifying the focusing parameter, *γ*, is much larger than that in modifying the weighting factor, *α*. The optimal *α* values are found in the range [0.25, 0.75], and the *α* = 0.5 value performs well in most cases. Similar to those findings, as in Table 4, we have empirically shown that the hyperparameter values *γ* = 2 and *α* = 0.5 produce the best classification performance for our HSI dataset. Although there is an extreme imbalance in the dataset, the same α value, which is 0.5, is selected for both positive and negative classes. The reason is that the easy positives are down-weighted with the help of *γ* and the negatives require less focus from the loss function. As a result, the model training concentrates on the hard examples rather than intentionally focusing on the minority class. The other α values, 0.25 and 0.5, still perform similarly for the same *γ* values. Therefore, we can conclude that the value of *γ* is the critical factor in the loss function, while the *α* parameter should be optimized for each *γ* value. In the CE configuration, the classifier outputs the lowest precision since its false positive rate is relatively high. When the *α* value is set to 0.25 in BCE, we see a significant improvement in precision thanks to the drop in false positives. We can confirm that *α* plays an important role in identifying the cost function behavior in BCE form. Nevertheless, the FL function performs much better than the BCE function since it can force the learner to focus on hard examples independent from the class label.

A further experiment we conducted compared the classification performance for different spectral resolutions. For this purpose, we compared a hyperspectral dataset (HSI), sampled hyperspectral datasets (HSI-90, HSI-30, and HSI-10), two PCA-based versions of the hyperspectral dataset (PCA-9 and PCA-3), and RGB versions of our dataset. The initial HSI dataset consists of 270 bands. By sampling individual bands from the HSI dataset with a constant frequency, we have generated 90 (HSI-90)-, 30 (HSI-30)-, and 10 (HSI-10)-band versions of the initial dataset. Additionally, we have applied dimensionality reduction to our HSI dataset with the help of the PCA method. We have utilized the PCA algorithm presented in [38], an incremental technique to calculate the PCA of large datasets. We selected the first nine principal components using a variance threshold value of 0.1%. We have also formed another PCA dataset by using the first three principal components to do a three-bands performance comparison with the RGB dataset. The first three principal components (PCA-3) had a total variance of 93.46%, and the first nine principal components (PCA-9) had a total variance of 98.60%. In addition to hyperspectral datasets, we have employed the RGB data simultaneously captured by our hyperspectral camera with HSI data. The RGB data captured by the camera contains three individual bands taken from the 630, 540, and 480 nm wavelengths, respectively. We have used the RGB images to train another 3D-CNN model with the same topology. For supporting three channels input to our 3D-CNN learner, we have set convolution kernel depth and stride parameters accordingly and kept the other parameters the same as in its original version. As shown in Table 5, Table 6, Table 7, Table 8, Table 9 and Table 10, the focal loss function hyperparameters of 3D-CNN for the datasets are fine-tuned empirically. According to the experimentation results, HSI performs the best with the highest accuracy and MCC score. The results of the sampled HSI datasets clearly show that more hyperspectral bands result in higher classification performance. For the sampled hyperspectral datasets, the classification accuracy is directly proportional to the number of bands contained in the dataset. The PCA-9 dataset has the second-best classification accuracy since it holds most of the variance from the original HSI dataset. The PCA-3 dataset has a lower accuracy than PCA-9, but a higher accuracy and MCC score than the RGB dataset.

In our fourth experiment, we compared the implications of convolution operation on classification performance. For this purpose, instead of a 3D convolution operation, we trained another classification model with a 2D convolution operation with the same network topology. We fine-tuned the focal loss hyperparameters for the 2D convolution case as given in Table 11. We found that the hyperparameter set, *γ* = 2 and *α* = 0.5, that was best with 3D-CNN was also best for the 2D-CNN model. Two-dimensional convolution operates in two directions of the image data, whereas 3D convolution slides in three directions of the hyperspectral cube. Therefore, the descriptive power of the feature sets collected by 2D and 3D convolution operations are different. As depicted in Table 12, the 3D convolution operator performed better than the 2D version. From the analysis, we infer that the 3D convolution operator can utilize the full potential of hyperspectral data while the 2D convolution operator causes a deterioration of classification performance for hyperspectral data.

In our fifth experiment setup, we showed that our model is free from overfitting by rotating the split sets, training, validation, and testing, between each other. There are 3 healthy and 27 unhealthy patients in our whole dataset. We created three different data-splitting configurations by putting one healthy and nine unhealthy patients in each of the training, validation, and testing sets. We rotated the sets between each other and repeat model training for each configuration. As shown in Table 13, we obtained similar classification performance results for all three configurations. From this experiment, we empirically show that our 3D-CNN model is capable of learning descriptive features from hyperspectral space without overfitting the given training data.

## 4. Discussions and Conclusions

In this study, we have proposed a new HCC tumor detection method utilizing hyperspectral imaging and a custom deep-learning model. We have built a biological tissue imaging system by integrating a VNIR hyperspectral camera with a light microscopy device. We collected hyperspectral images of tumor and healthy liver tissues with the help of our imaging system. We have designed a custom 3D-CNN classification topology to utilize the full potential of HSI data. In our CNN topology, we have included four convolution layers with max-pooling layers between them. The max-pooling layers down-sample the data by halving the size of the dataset at every iteration, effectively reducing model complexity. The use of 3D convolution layers enables us to leverage both textural and spectral features in a single training pipeline. Moreover, our method does not require separate feature extraction operations on the dataset, and the learner can automatically extract useful features from the training set. In addition to 3D convolutions employed in the deep learning model, we have optimized the network topology by replacing the traditional cross-entropy cost function with the focal loss cost function. In this way, we have significantly overcome the class imbalance problem residing in our dataset. The focal loss function made the classification model less biased towards the majority class (unhealthy). Well-classified easy examples are down-weighted with the help of the focal loss function; thus, the training procedure concentrates on learning hard examples. We have empirically optimized the hyperparameters of the focal loss function, *γ* and *α*, for each experiment configuration. Notably, the *γ* parameter in the focal loss function has a critical impact on the classification performance whereas α has a minor effect on the results.

The majority of computer-aided histopathology studies rely on RGB data captured with a Complementary Metal-Oxide Semiconductor (CMOS) or CCD sensors [39,40,41]. Our study utilizes a much wider range of the electromagnetic spectrum. The hyperspectral dataset used in our study includes contiguous 270 bands between 400 to 800 nm in the spectrum, whereas the RGB dataset includes three individual bands taken from 630, 540, and 480 nm. With the help of hyperspectral imaging, the subject material’s chemical composition can be analyzed in addition to conventional spatial attributes such as size, shape, and texture. The hyperspectral cube is versatile for our classification task since it can represent the variation of material properties in fine detail as spectral signatures. Unlike an RGB dataset, the descriptive features along the spectral dimension can be effectively captured by a 3D convolution operation. Our 3D-CNN-based supervised learner can describe the nonlinear relationships between the features in both spectral and spatial dimensions. That is, features such as corners, edges, and textures in the spatial plane can be associated with features such as peaks, dips, slopes, and valleys in the spectral signatures of pixels. The large amount of information within the hyperspectral cube enables the deep learning model to build a strong classifier with highly descriptive feature extraction competency. Moreover, by sampling the original hyperspectral dataset into lower dimension datasets such as HSI-90, HSI-30, and HSI-10, we observe the advantage of having more bands in classification. In other words, the deep learning model’s prediction power is enhanced by introducing more spectral bands to the learner. Additionally, we have used PCA for dimensionality reduction on the original hyperspectral data with 270 bands. We have generated two other datasets with, first, nine principal components (PCA-9) and then three principal components (PCA-3). The PCA method significantly reduces data complexity and improves the signal-to-noise ratio; hence it becomes easier for the learner to converge. However, the CNN models trained with PCA data yielded lower classification accuracy than the CNN model trained with HSI data. The PCA-9 dataset having a maximum variance of 98.60% performed almost as well as the HSI dataset. Considering the simplicity of PCA-9 compared to the original HSI dataset, PCA provides a cost-effective way of utilizing hyperspectral data for our task. The PCA-3 dataset performed better than the RGB dataset, indicating that hyperspectral data compressed into three bands contains more useful information for classifying tissue samples than RGB. Experimental results validate the resourcefulness of the HSI dataset over its RGB and PCA counterparts on classification accuracy.

Although we have proposed a 3D-CNN classification model with promising results, there are limitations to our study. Firstly, the dataset employed in the study needs to be extended by adding more tissue samples. With more data fed into the training stage, the resultant classifier is expected to be more resistant to the overfitting phenomenon and have a better generalization capability. It is desirable to assess our model with further validation with a larger tissue sample dataset collected from various laboratories labeled by different pathologists. This way, the dataset’s sample variation can be boosted, and the classification model can span a larger area in feature space.

In summary, the model can be used for supporting pathologists’ examination or initial screening. Our methodology can serve as a decision support tool for novice pathologists even though the model does not provide a holistic tissue examination, including inspection of inflammation, necrosis, and blood vessels, as pathologists do.

## Figures and Tables

**Table 1:** 3D-CNN Topology with parameters.

Layer	Parameters	Output Size
Input		100 × 100 × 270
Convolution3D	Kernel Size: 3 × 3 × 3 Number of filters: 4 Activation: ReLU Padding: same	100 × 100 × 270 × 4
Max-pooling3D	Pool Size: 3 × 3 × 3 Strides: 2 × 2 × 2 Padding: same	50 × 50 × 135 × 4
BatchNormalization		50 × 50 × 135 × 4
Convolution3D	Kernel Size: 3 × 3 × 3 Number of filters: 8 Activation: ReLU Padding: same	50 × 50 × 135 × 8
Max-pooling3D	Pool Size: 3 × 3 × 3 Strides: 2 × 2 × 2 Padding: same	25 × 25 × 68 × 8
BatchNormalization		25 × 25 × 68 × 8
Convolution3D	Kernel Size: 3 × 3 × 3 Number of filters: 16 Activation: ReLU Padding: same	25 × 25 × 68 × 16
Max-pooling3D	Pool Size: 3 × 3 × 3 Strides: 2 × 2 × 2 Padding: same	13 × 13 × 34 × 16
BatchNormalization		13 × 13 × 34 × 16
Convolution3D	Kernel Size: 3 × 3 × 3 Number of filters: 32 Activation: ReLU Padding: same	13 × 13 × 34 × 32
Max-pooling3D	Pool Size: 3 × 3 × 3 Strides: 2 × 2 × 2 Padding: same	7 × 7 × 17 × 32
BatchNormalization		7 × 7 × 17 × 32
GlobalAveragePooling3D		32
Dense	Units: 512 Activation: ReLU	512
Dropout	Drop rate: 0.1	512
Dense (Classification)	Units: 1	1

**Table 2:** Dataset statistics with the class distribution.

	Healthy	Unhealthy	Total
Training Samples	2	18	20
Training Cases	1	9	10
Validation Samples	2	18	20
Validation Cases	1	9	10
Testing Samples	2	18	20
Testing Cases	1	9	10
**Total Samples**	6	54	60
**Total Cases**	3	27	30

**Table 3:** Classification results for varying patch size parameter. (focusing parameter γ = 2, weighting factor α = 0.50, 3D convolutions, see Figure A1 for network topology).

Patch Size (S)	Accuracy	Precision	Recall	F1-Score	MCC
50 × 50	0.929	0.996	0.924	0.961	0.722
100 × 100	0.970	0.999	0.968	0.984	0.860
150 × 150	0.961	0.973	0.983	0.967	0.774
200 × 200	0.924	0.968	0.947	0.945	0.615

**Table 4:** Classification results with HSI dataset for varying cost functions and respective parameter sets. (patch size parameter *S* = 100, 3D convolutions, see Figure A1 for network topology).

Loss Function	Accuracy	Precision	Recall	F1-Score	MCC
CE	0.891	0.921	0.961	0.906	0.277
BCE (γ=0, α=0.25)	0.901	0.938	0.953	0.919	0.412
BCE (γ=0, α=0.75)	0.902	0.916	0.981	0.909	0.278
FL (γ=1.5, α=0.25)	0.918	0.982	0.926	0.949	0.646
FL (γ=1.5, α=0.50)	0.922	0.978	0.935	0.949	0.643
FL (γ=1.5, α=0.75)	0.930	0.969	0.952	0.949	0.638
FL (γ=2.0, α=0.25)	0.960	0.993	0.962	0.976	0.811
FL (γ=2.0, α=0.50)	0.970	0.999	0.968	0.984	0.860
FL (γ=2.0, α=0.75)	0.955	0.983	0.966	0.969	0.766
FL (γ=2.5, α=0.25)	0.948	0.998	0.943	0.972	0.782
FL (γ=2.5, α=0.50)	0.958	0.999	0.955	0.978	0.818
FL (γ=2.5, α=0.75)	0.953	0.986	0.962	0.969	0.768

**Table 5:** Classification results with HSI-90 dataset for varying cost functions and respective parameter sets. (patch size parameter *S* = 100, 3D convolutions, see Figure A2 for network topology).

Loss Function	Accuracy	Precision	Recall	F1-Score	MCC
FL (γ=1.5, α=0.25)	0.885	0.973	0.897	0.927	0.539
FL (γ=1.5, α=0.50)	0.892	0.975	0.903	0.932	0.559
FL (γ=1.5, α=0.75)	0.897	0.971	0.913	0.933	0.554
FL (γ=2.0, α=0.25)	0.919	0.980	0.929	0.949	0.645
FL (γ=2.0, α=0.50)	0.950	0.990	0.955	0.970	0.767
FL (γ=2.0, α=0.75)	0.924	0.986	0.929	0.954	0.679
FL (γ=2.5, α=0.25)	0.914	0.977	0.926	0.944	0.619
FL (γ=2.5, α=0.50)	0.885	0.977	0.894	0.929	0.552
FL (γ=2.5, α=0.75)	0.857	0.970	0.868	0.91	0.473

**Table 6:** Classification results with HSI-30 dataset for varying cost functions and respective parameter sets. (patch size parameter *S* = 100, 3D convolutions, see Figure A2 for network topology).

Loss Function	Accuracy	Precision	Recall	F1-Score	MCC
FL (γ=1.5, α=0.25)	0.877	0.970	0.890	0.921	0.510
FL (γ=1.5, α=0.50)	0.885	0.973	0.897	0.927	0.537
FL (γ=1.5, α=0.75)	0.885	0.971	0.90	0.926	0.53
FL (γ=2.0, α=0.25)	0.889	0.974	0.900	0.930	0.55
FL (γ=2.0, α=0.50)	0.931	0.985	0.937	0.957	0.692
FL (γ=2.0, α=0.75)	0.909	0.975	0.923	0.941	0.598
FL (γ=2.5, α=0.25)	0.883	0.971	0.897	0.925	0.524
FL (γ=2.5, α=0.50)	0.861	0.973	0.870	0.914	0.495
FL (γ=2.5, α=0.75)	0.857	0.97	0.868	0.91	0.473

**Table 7:** Classification results with HSI-10 dataset for varying cost functions and respective parameter sets. (patch size parameter *S* = 100, 3D convolutions, see Figure A2 for network topology).

Loss Function	Accuracy	Precision	Recall	F1-Score	MCC
FL (γ=1.5, α=0.25)	0.857	0.967	0.871	0.909	0.46
FL (γ=1.5, α=0.50)	0.857	0.970	0.868	0.910	0.472
FL (γ=1.5, α=0.75)	0.859	0.966	0.874	0.909	0.46
FL (γ=2.0, α=0.25)	0.914	0.978	0.926	0.945	0.622
FL (γ=2.0, α=0.50)	0.910	0.977	0.922	0.942	0.61
FL (γ=2.0, α=0.75)	0.898	0.973	0.911	0.934	0.565
FL (γ=2.5, α=0.25)	0.867	0.973	0.876	0.917	0.505
FL (γ=2.5, α=0.50)	0.862	0.973	0.871	0.914	0.498
FL (γ=2.5, α=0.75)	0.890	0.970	0.905	0.928	0.534

**Table 8:** Classification results with PCA-9 dataset for varying cost functions and respective parameter sets. (patch size parameter *S* = 100, 3D convolutions, see Figure A2 for network topology).

Loss Function	Accuracy	Precision	Recall	F1-Score	MCC
FL (γ=1.5, α=0.25)	0.897	0.975	0.909	0.934	0.571
FL (γ=1.5, α=0.50)	0.911	0.977	0.923	0.943	0.614
FL (γ=1.5, α=0.75)	0.911	0.974	0.926	0.941	0.602
FL (γ=2.0, α=0.25)	0.945	0.987	0.952	0.966	0.743
FL (γ=2.0, α=0.50)	0.957	0.988	0.964	0.972	0.788
FL (γ=2.0, α=0.75)	0.927	0.987	0.932	0.956	0.687
FL (γ=2.5, α=0.25)	0.930	0.980	0.941	0.954	0.674
FL (γ=2.5, α=0.50)	0.892	0.981	0.897	0.934	0.583
FL (γ=2.5, α=0.75)	0.882	0.976	0.891	0.927	0.544

**Table 9:** Classification results with PCA-3 dataset for varying cost functions and respective parameter sets. (patch size parameter *S* = 100, 3D convolutions, see Figure A2 for network topology).

Loss Function	Accuracy	Precision	Recall	F1-Score	MCC
FL (γ=1.5, α=0.25)	0.873	0.971	0.885	0.919	0.503
FL (γ=1.5, α=0.50)	0.879	0.970	0.894	0.922	0.511
FL (γ=1.5, α=0.75)	0.857	0.941	0.897	0.897	0.336
FL (γ=2.0, α=0.25)	0.899	0.977	0.910	0.936	0.584
FL (γ=2.0, α=0.50)	0.913	0.983	0.919	0.947	0.638
FL (γ=2.0, α=0.75)	0.906	0.975	0.920	0.939	0.590
FL (γ=2.5, α=0.25)	0.888	0.976	0.897	0.930	0.556
FL (γ=2.5, α=0.50)	0.889	0.974	0.900	0.930	0.550
FL (γ=2.5, α=0.75)	0.923	0.972	0.942	0.947	0.626

**Table 10:** Classification results with RGB dataset for varying cost functions and respective parameter sets. (patch size parameter *S* = 100, 3D convolutions, see Figure A2 for network topology).

Loss Function	Accuracy	Precision	Recall	F1-Score	MCC
FL (γ=1.5, α=0.25)	0.852	0.964	0.868	0.905	0.440
FL (γ=1.5, α=0.50)	0.851	0.966	0.865	0.905	0.449
FL (γ=1.5, α=0.75)	0.828	0.936	0.869	0.879	0.269
FL (γ=2.0, α=0.25)	0.900	0.974	0.914	0.936	0.573
FL (γ=2.0, α=0.50)	0.891	0.972	0.905	0.930	0.544
FL (γ=2.0, α=0.75)	0.882	0.970	0.897	0.924	0.520
FL (γ=2.5, α=0.25)	0.859	0.970	0.871	0.911	0.479
FL (γ=2.5, α=0.50)	0.853	0.968	0.865	0.907	0.458
FL (γ=2.5, α=0.75)	0.878	0.964	0.897	0.919	0.486

**Table 11:** Classification results with HSI dataset for varying cost functions and respective parameter sets. (patch size parameter *S* = 100, 2D convolutions, see Figure A3 for network topology).

Loss Function	Accuracy	Precision	Recall	F1-Score	MCC
FL (γ=1.5, α=0.25)	0.891	0.974	0.903	0.931	0.554
FL (γ=1.5, α=0.50)	0.885	0.972	0.897	0.926	0.534
FL (γ=1.5, α=0.75)	0.885	0.970	0.900	0.926	0.527
FL (γ=2.0, α=0.25)	0.922	0.983	0.929	0.952	0.663
FL (γ=2.0, α=0.50)	0.934	0.986	0.940	0.959	0.706
FL (γ=2.0, α=0.75)	0.920	0.980	0.930	0.949	0.646
FL (γ=2.5, α=0.25)	0.932	0.983	0.94	0.957	0.689
FL (γ=2.5, α=0.50)	0.923	0.982	0.931	0.952	0.658
FL (γ=2.5, α=0.75)	0.915	0.975	0.929	0.944	0.615

**Table 12:** Comparison of classification results of 3D-CNN and 2D-CNN models trained by HSI data.

Model	Accuracy	Precision	Recall	F1-Score	MCC
HSI-3D-CNN	0.970	0.999	0.968	0.984	0.860
HSI-2D-CNN	0.934	0.986	0.940	0.959	0.706

**Table 13:** Comparison of classification results of 3D-CNN when the training, validation, and testing sets are rotated between each other. (patch size parameter *S* = 100, 3D convolutions, see Figure A1 for network topology).

Model	Accuracy	Precision	Recall	F1-Score	MCC
HSI-3D-CNN (configuration-1)	0.970	0.999	0.968	0.984	0.860
HSI-3D-CNN (configuration-2)	0.965	0.997	0.963	0.981	0.836
HSI-3D-CNN (configuration-3)	0.968	0.996	0.968	0.982	0.846

**Figure A1:**
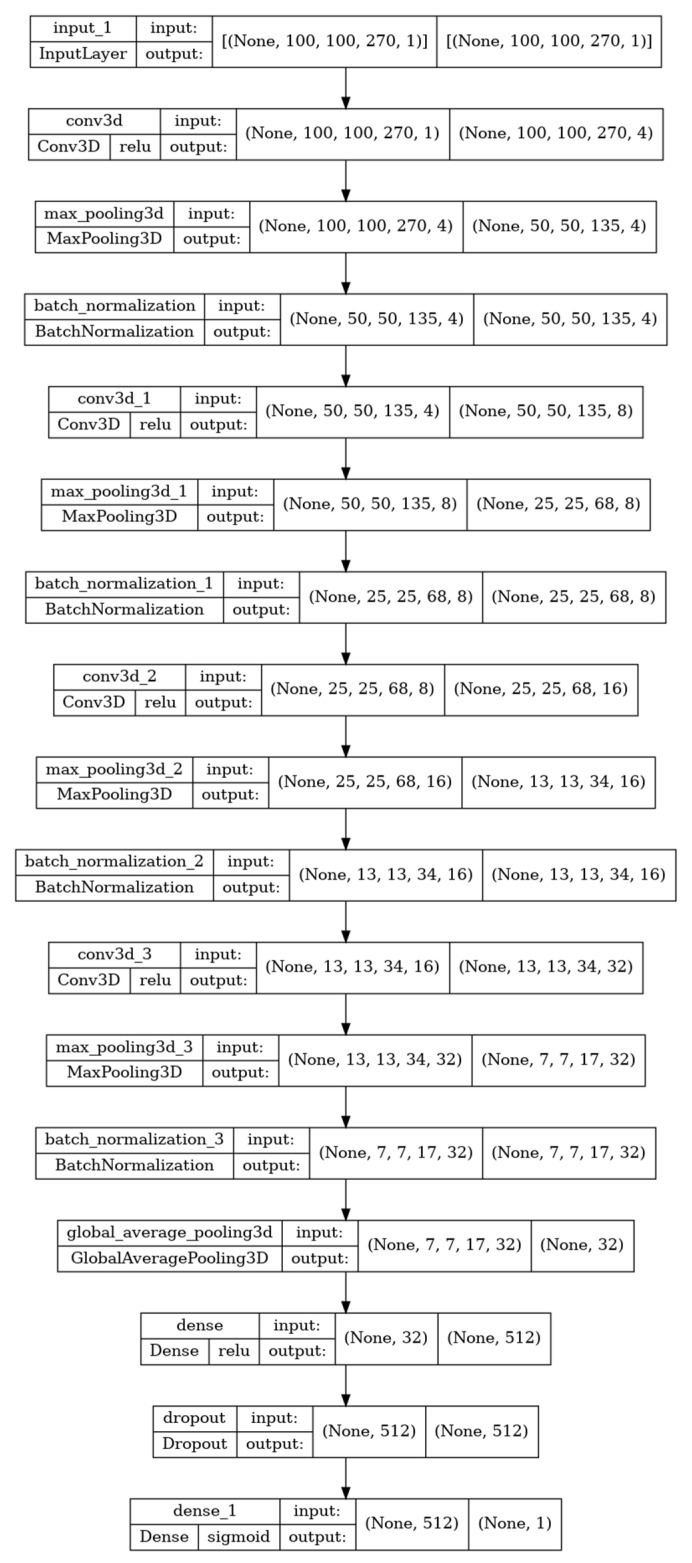
Topology of 3D-CNN utilized for HSI data.

**Figure A2:**
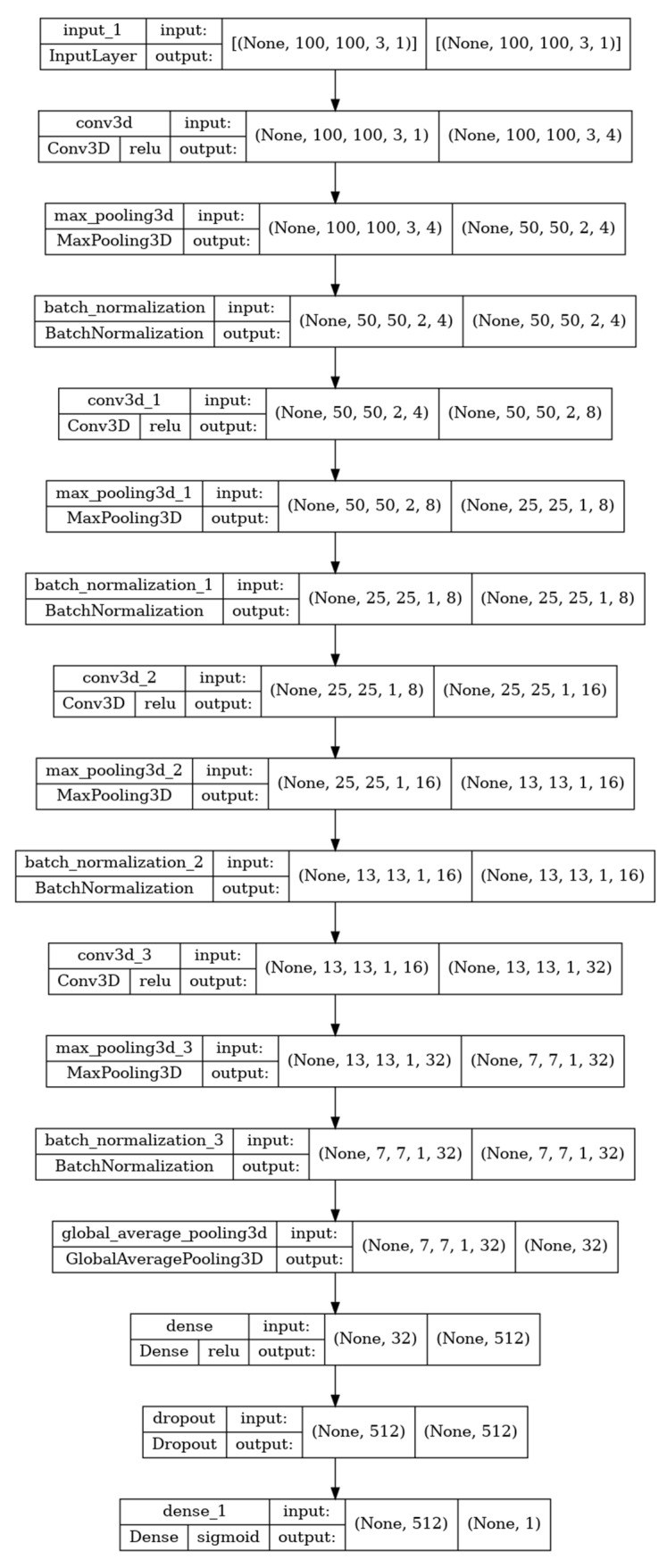
Topology of 3D-CNN utilized for Three-Channel data.

**Figure A3:**
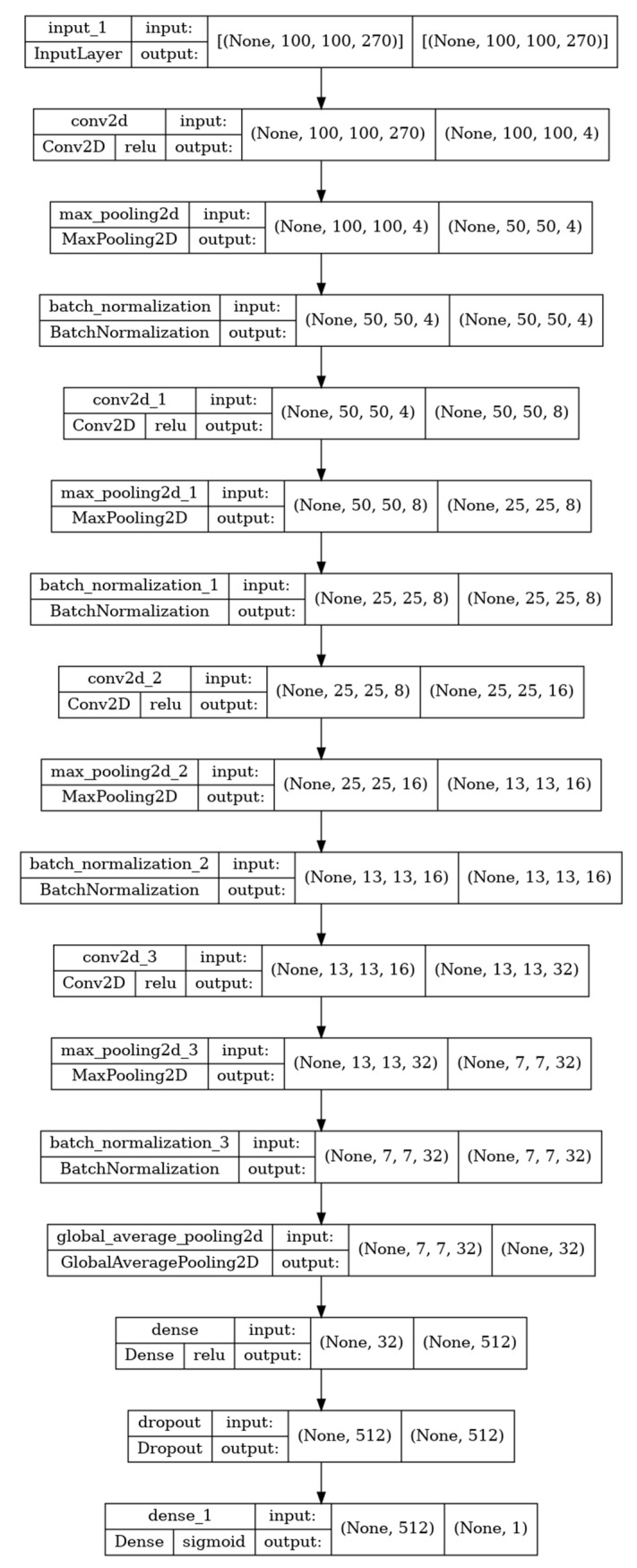
Topology of 2D-CNN utilized for HSI data.

## Data Availability

The data presented in this study are available on request from the corresponding author. The data are not publicly available as the intellectual property rights of the original tissue samples are owned by the microarray vendor.
